# Associations of quality of social support with affective symptoms from midlife to later life: evidence from a British birth cohort

**DOI:** 10.1017/S0033291726104930

**Published:** 2026-07-09

**Authors:** Alexandra Schmidt, Ellen J. Thompson, Marcus Richards, Clara Strauss, Nick Grey, Darya Gaysina

**Affiliations:** 1 University of Sussex, Brighton, UK; 2 University College London, UK; 3 Sussex Partnership NHS Foundation Trust, Worthing, West Sussex, UK

**Keywords:** affective symptoms, anxiety, birth cohort study, depression, later life, longitudinal, midlife, social support, growth mixture modelling, life course, ageing, mental health

## Abstract

**Background:**

Quality of social support is linked to mental health, but less is known about its long-term effects. We aimed to investigate the effects of the quality of social support on affective symptoms from midlife through later life.

**Methods:**

Data were used from the MRC National Survey of Health and Development (NSHD), a prospective birth cohort originally consisting of 5,362 people born in 1946. Affective symptoms were measured at ages 53, 60–64, and 69 years using the General Health Questionnaire (GHQ-28), and longitudinal affective symptom trajectories were derived using growth mixture modeling. Quality of social support (positive and negative) was assessed at age 53 years with an adapted version of the Close Persons Questionnaire. Associations of positive and negative social support with affective symptoms at each age and with the longitudinal trajectories were tested using structural equation modeling and the R3 Step approach.

**Results:**

Four distinct affective symptom trajectories were identified: no/low symptoms (83%), low and increasing symptoms (8%), consistently moderate/high symptoms (5%), and moderate/high and decreasing symptoms (4%). In fully adjusted models, negative social support was associated with affective symptoms at all three ages ( *β* : 0.09–0.16, all *p*-values < .001) and with the ‘consistently moderate/high symptoms’ trajectory (OR = 1.65, 95% CI: 1.36, 2.01, *p* < .001); no association was found for positive social support.

**Conclusions:**

Results highlight the importance of negative social support as a potential modifiable factor in prevention and intervention initiatives for affective symptoms among adults from midlife to later life.

Affective disorders, such as depression and anxiety, are common among adults (Baker & Kirk-Wade, 2024; Gondek et al., 2022), with one in six adults being affected (Santomauro et al., 2021). They are associated with increased comorbidity and mortality (Hare, Toukhsati, Johansson, & Jaarsma, 2014; Momen et al., 2022; Rodda, Walker, & Carter, 2011) and adversely impact quality of life (Sivertsen et al., 2015). Midlife has been postulated as a pivotal period in one’s life course, marking a point from which trajectories of growth or decline may emerge across many domains, including mental health (Lachman, Teshale, & Agrigoroaei, 2015). This period, therefore, provides potential for curtailing declining processes by addressing risk factors or employing protective resources (Lachman, Teshale, & Agrigoroaei, 2015).

Social support has a strong theoretical basis in the development of affective symptoms, for example, through the stress buffering hypothesis (Cohen & Wills, 1985), whereby social support can exert an overall beneficial effect on mental health or act as a buffering mechanism in times of adversity, which might otherwise have pathological effects; similarly, the ‘need to belong’ theory (Baumeister & Leary, 1995) postulates the basic human need for belongingness and interpersonal relationships and the associated potential maladaptive outcomes for those who lack these social bonds.

Different conceptualizations of social support exist and range from instrumental to emotional support, and can relate to quantity (e.g. social network size) rather than quality (Langford, Bowsher, Maloney, & Lillis, 1997). However, previous research indicated that the quality rather than quantity of social support was linked to better mental health outcomes (Benca-Bachman et al., 2020). The quality of social support encompasses positive aspects such as emotional support by close persons as well as negative aspects, such as conflict or stress, which could result from behaviors such as unwanted advice or intrusion, failure to provide needed help, unsympathetic or insensitive behavior, and rejection or neglect (Newsom et al., 2005). In the context of their effect on health, positive social support includes having companionship, feeling listened to, and sharing enjoyable activities. In contrast, negative social support may occur when there is a failure to provide needed support, provide grudging support, or failure to include a person in enjoyable shared activities (Rook, 2015). A recent study reported historical increases in loneliness among midlife adults compared to earlier-born cohorts (Infurna et al., 2025), making this an important area for consideration in the development of affective disorders.

A number of systematic literature reviews confirm associations of social support with depression (Gariépy, Honkaniemi, & Quesnel-Vallée, 2016; Maier, Riedel-Heller, Pabst, & Luppa, 2021) and anxiety (Zimmermann, Chong, Vechiu, & Papa, 2020). For example, Gariépy, Honkaniemi, and Quesnel-Vallée (2016) reviewed 100 studies and found consistent evidence for the role of social support as a protective factor against depression in ‘younger’ adults (< 50 years) and ‘older’ adults (50+ years). However, a considerable number of eligible studies in the above mentioned reviews included younger adults (Kendler, Myers, & Prescott, 2005; Vriends, Becker, Meyer, & Margraf, 2011) or older adults (65+ years) only (Harris et al., 2006), were cross-sectional, or had short follow-up times (< 5 years) (Cacioppo et al., 2006; Stafford, McMunn, Zaninotto, & Nazroo, 2011), making it difficult to draw conclusions on longitudinal associations of social support with affective symptoms starting from midlife.

Moreover, there is limited evidence on how social support is related to longitudinal trajectories of affective symptoms. Longitudinal trajectories of affective symptoms are not homogeneous and may vary in onset, severity, and longevity (Bromberger et al., 2019; Musliner, Munk-Olsen, Eaton, & Zandi, 2016). Therefore, the aim of this study was to investigate the longitudinal association of positive and negative social support at midlife (age 53 years) with affective symptoms over the period of two decades, specifically the objectives were: (1) to derive affective symptom trajectories from midlife (age 53 years) to later life (age 69 years), and (2) to investigate longitudinal associations of quality of social support at midlife with affective symptoms at ages 53, 60–64, and 69 years, and with affective symptom trajectories.

## Methods

### Participants

The study used data from the MRC National Survey of Health and Development (NSHD). The original sample comprised 5,362 males and females born during 1 week in March 1946 across England, Scotland, and Wales. The cohort has been followed prospectively 25 times across their life course from birth until the most recent data collection in 2020, when study members were 74 years old (Kuh et al., 2016; Wadsworth, Kuh, Richards, & Hardy, 2006). In this study, we will primarily focus on data from ages 53, 60–64, and 69.

Participants gave signed informed consent to participate in the NSHD study (D. Kuh et al., 2011), and ethical approval was granted by the National Research Ethics Service Committee London Queen Square (14/LO/1073) and by the Scotland A Research Ethics Committee (14/SS/1009). The current study received ethical approval from the University of Sussex’s Departmental Ethics Board (ER/AS2085/4).

### Measures

#### Affective symptoms

Affective symptoms were assessed at ages 53, 60–64, and 69 via the 28-item version of the General Health Questionnaire (GHQ-28) (Goldberg & Hillier, 1979). The GHQ-28 is a scaled questionnaire designed to measure psychological distress. It comprises four different subscales with seven items each: somatic symptoms, anxiety and insomnia, social dysfunction, and severe depression (Supplementary Appendix G). Cohort members were asked to respond on a scale from four possible answers: ‘not at all’, ‘no more than usual’, ‘rather more than usual’, and ‘much more than usual’, scored from 0 to 3. Upon inspection, the data were positively skewed, with most cohort members’ answers in the first two categories, which resulted in low cell counts in the last two categories. Therefore, the last two categories were collapsed into a single category of ‘rather more or much more than usual’, resulting in item coding 0-1-2-2. The GHQ has previously been reported as a consistent and reliable measure for the detection of affective symptoms in the general population with long intervals between applications (Pevalin, 2000). In this study, internal consistency was assessed using Cronbach’s alpha for comparability with other studies and was good with values ranging from .90 for ages 60–64 and 69 years to .92 for age 53 years. Given evidence for a multidimensional structure and in line with recommendations by Yang and Green (2011), omega coefficients derived from the bifactor model were also calculated and indicated strong reliability of the total score and substantial saturation by the general factor for age 53 (*ω* .97; *ωH* = .86), age 60–64 (*ω* .97; *ωH* = .84), and age 69 (*ω* .97; *ωH* = .84).

#### Positive and negative social support

An adapted version of the Close Persons Questionnaire (Stansfeld & Marmot, 1992) was completed at age 53. The questionnaire asked cohort members to nominate the person they felt closest to in the last 12 months and answer six questions about the quality of that relationship. Example items include ‘How much in the last 12 months did this person make you feel good about yourself?’ and ‘How much in the last 12 months would you have liked to have confided more in this person?’, which could be answered on a 4-point scale, ranging from 0 = ‘not at all’ to 3 = ‘a great deal.’ The hypothesized two-factor model showed good fit to the data: *χ^2^* (15) = 2839.36, *p* < .001, CFI = .98, TLI = .96, RMSEA = .051 (90% CI: [.040, .063]), SRMR = .027. All items loaded significantly on their intended factors (standardized loadings = .50–.82). Based on this, positive and negative social support scores were derived with a possible range from 0 to 9 for each. The Close Persons Questionnaire has shown good reliability and validity in previous research (Hanssen et al., 2019). Cronbach’s alpha ( *α* ) in this study was .73 for positive social support and .60 for negative social support.

#### Covariates

Based on previous research, we adjusted for factors known to be associated with both affective symptoms and social support, namely sex (Milner, Krnjacki, & LaMontagne, 2016), education (Amin, Fletcher, Lu, & Song, 2023), social class (Lang et al., 2011), marital status (Schoevers et al., 2000), physical health problems (Jones, Minarik, Gilliss, & Lee, 2020), and previous mental health problems in order to account for potential confounding and to reduce the likelihood of reverse directionality (Lyness et al., 2009). Educational level was based on the highest attainment at age 26 years and was categorized as: no qualification, below secondary qualification, secondary qualification (‘O’ levels or training equivalents), advanced secondary qualification (‘A’ levels or equivalents), or higher education (degree level or equivalent). Social class at age 53 was categorized as: professional, intermediate, skilled (nonmanual), skilled (manual), partly skilled, and unskilled. Physical health problems were identified by asking cohort members if, in the last 12 months, they had developed a serious illness or disability with a no/yes answer. Previous mental health problems were measured via the teacher-rated forerunner of the Rutter A scale at age 15 years (Rutter, 1967), and via the shortened version of the Present State Examination at age 36 years (Wing, Cooper, & Sartorius, 1974).

### Statistical analysis

#### Confirmatory factor analysis (CFA)

A CFA in bifactor models was conducted on GHQ-28 measures at ages 53, 60–64, and 69 to estimate single latent factor scores of affective symptoms at each time point, using the WLSMV estimator. In the bifactor model, every item in the scale is influenced by two factors: a global factor of affective symptoms, which comprises the variance shared by all items; and a group factor, accounting for any residual common variance of specific items associated with the group factors (GHQ-28 four subscales: somatic, anxiety/insomnia, social dysfunction, and severe depression). Measures of *χ* ^2^ are sensitive to sample size and need to be interpreted with caution; therefore, other fit indices were also considered (Marsh, Balla, & McDonald, 1988). The Comparative Fit Index (CFI) and the Tucker–Lewis Index (TLI) range from 0 to 1, with values >.95 reflecting an excellent fit and .90 reflecting an acceptable fit to the data. Root mean square error of approximation (RMSEA) values of < .05 and <. 08 represent a close and acceptable fit to the data, respectively (Browne & Cudeck, 1992; Fan, Thompson, & Wang, 1999; Hu & Bentler, 1999). The derived factor scores were used as continuous variables of affective symptoms at each age.

#### Trajectory class models

Initially, measurement invariance was tested to ensure that the same latent construct is tested at all time points (Supplementary Appendix B provides fit indices for the measurement invariance tests). As *χ* ^2^ tests are sensitive to sample size, alternative fit indices were also checked. Recommended cutoffs for achieving measurement invariances: for RMSEA, the change is ≥0.010; for SRMR, the change is ≤0.03; and for CFI, the change is ≤0.01 (Chen, 2007; Cheung & Rensvold, 2002).

As a first step, a latent growth curve analysis was conducted to fit an average latent growth curve. As this analysis includes measurements from only three time points, it was restricted to a linear growth curve. The latent growth curve includes an estimated mean intercept and an estimated mean slope, and this model depicts the pattern of affective symptom change for all participants as a homogeneous group. Next, growth mixture models (GMMs) were fitted to identify different numbers of latent class trajectories, starting with a model with two classes, up to a five-class model. Each model was compared to a model with one fewer class, using model fit indicators Akaike’s Information Criteria (AIC), Bayesian Criterion Information (BIC), Vuong-Lo-Mendel-Rubin adjusted likelihood ratio test (VMLR-LRT), the bootstrap likelihood ratio test (BLRT), entropy, and the sample size in each class. Lower AIC or BIC values indicate a better fit. A significant *p*-value (<.05) for the VLMR-LRT or BLRT indicates that the current (more complex) model provides a better fit than a model with one fewer class. In case of discrepancy between indices, models with the lowest BIC have been favored in the literature (van de Schoot et al., 2017). Entropy values (ranging from 0 to 1) were examined to see if participants fitted well into the created classes. Higher entropy values indicate a higher level of accuracy of participant classification, with > .80 high class separation, 0.6 medium, and 0.4 low (Celeux & Soromenho, 1996). As per the recommendation by Jung and Wickrama (2008), we preferred models where each class contained no less than 1% of the total sample.

All models were run using the full-information maximum likelihood (FIML) estimation with robust standard errors (MLR), which draws on all available data points in the dataset and includes all individuals who had at least one of the three measures of affective symptoms (*n* = 3,121).

#### Associations of social support with affective symptoms

First, we tested for associations between negative and positive social support and affective symptoms measured with latent variables at ages 53, 60–64, and 69, using a structural equation modeling (SEM) framework. Three SEMs were run: Model 1 with positive and negative social support separately, Model 2 with positive and negative social support, mutually adjusted, and Model 3 with Model 2 additionally adjusted for covariates, including sex, nominated close person, marital status, limiting illness/disability, education, social class, and previous mental health problems. In Model 2, we additionally tested for moderation by sex by testing differences between models with constrained and unconstrained paths for males and females.

Second, associations between positive and negative social support and affective symptom trajectories were tested using the R3 Step approach with predictors as auxiliary variables. For each analysis, the ‘no/low affective symptoms’ trajectory was used as the reference group. Three models were run: Model 1 with positive and negative social support separately, Model 2 with positive and negative social support, mutually adjusted, and Model 3 with Model 2 additionally adjusted for covariates, including sex, nominated close person, marital status, limiting illness/disability, education, social class, and previous mental health problems. In Model 2, we additionally tested for moderation by sex.

To handle missing data in the predictor and covariate variables, we ran multiple imputation in Mplus, specifying 50 imputed datasets with 50 iterations (Austin, White, Lee, & van Buuren, 2021). We used Mplus version 8.10 (Muthen, Muthen, & Muthén, 2017) alongside R version 3.6.2 (R Core Team, 2020).

The Open Science Framework preregistration of the study can be found here: https://doi.org/10.17605/OSF.IO/9NPUM.

## Results

### Missing data and sample descriptives

Descriptive statistics for the sample of complete cases (*n* = 1,948) and imputed cases (*n* = 3,121) are presented in Table 1. Of the participants who had at least one measure of affective symptoms and for whom trajectories could be derived (*n* = 3,121), those with complete information on all predictors and covariates (*n* = 1,948) were compared to those with missing data (*n* = 1,173). Participants with missing data showed no significant difference in negative social support, marginally significant lower positive social support (*p* = .04, *d* = −0.08), and significantly higher mental health problems at age 36 (*p* = .03, *d* = 0.09) but not at age 15 years. There were no significant differences in sex, education, social class, or having an illness, but participants with missing data were less likely to be married and more likely to be single than those with complete data (*p* < .001).

**Table 1.** Descriptive statistics for complete sample (*N* = 1,948) and imputed sample (*N =* 3,121) Table 1. long description.

Variable	Level	Complete cases sample	Imputed sample
Positive social support, mean (SD)		6.47 (1.81)	6.43 (1.87)
Negative social support, mean (SD)		1.80 (1.61)	1.83 (1.64)
Sex, *n* (%)	Male	981 (50.4)	1,553 (49.8)
	Female	967 (49.6)	1,568 (50.2)
Nominated close person, *n* (%)	Spouse/partner	1,648 (84.6)	2,589 (82.9)
	Son/daughter	150 (7.7)	251 ((8.0)
	Other relative	77 (4.0)	145 (4.6)
	Friend or neighbor	66 (3.4)	115 (3.7)
	Other	7 (0.4)	21 (0.7)
Education, *n* (%)	No qualification	904 (46.4)	1,491 (47.8)
	Below ordinary secondary education	157 (8.1)	233 (7.5)
	Ordinary secondary education	489 (25.1)	767 (24.6)
	Advanced secondary education	233 (12.0)	360 (11.5)
	Higher education	165 (8.5)	270 (8.7)
Marital status, *n* (%)	Single	82 (4.2)	183 (5.9)
	Married/cohabiting	1,568 (80.5)	2,436 (78.1)
	Married/separated	43 (2.2)	71 (2.3)
	Divorced	204 (10.5)	343 (11)
	Widowed	51 (2.6)	88 (2.8)
Illness, *n* (%)	No	1,834 (94.1)	2,931 (93.9)
	Yes	114 (5.9)	190 (6.1)
Previous mental health problems, mean (SD)	Age 15 (0–4)	0.66 (1.00)	0.73 (1.07)
Previous mental health problems, mean (SD)	Age 36 (0–18)	2.18 (3.52)	2.34 (3.69)
Affective symptoms trajectories, *n* (%)	No/low symptoms	1,616 (83.0)	2,596 (83.2)
	Low and increasing	154 (7.9)	251 (8.0)
	Consistently moderate/high	97 (5.0)	164 (5.3)
	Moderate/high and decreasing	81 (4.2)	110 (3.5)

### Affective symptoms, latent factor scores, and longitudinal trajectories

#### Confirmatory factor analyses (CFAs)

CFAs were conducted to derive latent factor variables for the different ages using a bifactor model. The fit indices for this model are presented in Supplementary Appendix A, and the factor loadings in Supplementary Appendix G. CFI and TLI were excellent for all time points (CFI and TLI > .95). The root mean squared error of approximation (RMSEA) and the standardized root mean squared error (SRMSE) were acceptable with values below .08 for all time points. For the purposes of this study, we will focus on the global factor of affective symptoms.

#### Measurement invariance

The test for measurement invariance included the creation of a configural model and a scalar model. As this CFA model included ordered categorical variables and the metric of the factors was set by fixing the factor variance to one, a metric model was not created (Muthen, Muthen, & Muthén, 2017). The configural model fitted the data well, with all fit indices within the expected ranges. Next, to test for scalar invariance, factor loadings and thresholds were constrained to be equal across the time points. The model showed a good fit (Supplementary Appendix B), with all indicators supporting scalar invariance, indicating that the GHQ measured the same latent construct over time.

#### Latent class trajectories

**Figure 1.**
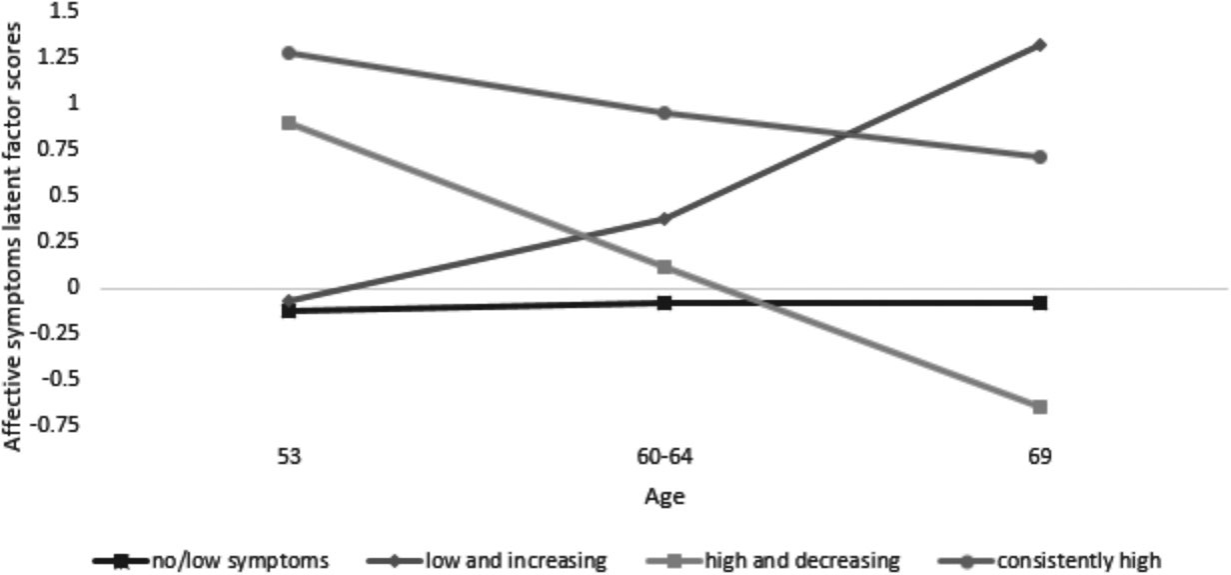
Longitudinal trajectories of affective symptoms from age 53 to 69 (*n* = 3,121). Figure 1. long description.

The linear latent growth curve analysis with one single average trajectory indicated that, on average, participants showed consistently low symptoms over time. We then modeled between two and five latent class trajectories using GMM to determine the number of classes. The four-class solution was selected as the best-fitting solution based on fit indicators and sample proportion in each class (see Table 2). Figure 1 shows the four affective symptom trajectories: low/no symptoms (*n* = 2,596, 83%), low and increasing (*n* = 251, 8%), consistently moderate/high (*n* = 164, 5%), moderate/high and decreasing (*n* = 110, 4%).

**Table 2.** Model fit indices for different affective symptom latent classes (*N* = 3,121) Table 2. long description.

	2 classes	3 classes	4 classes	5 classes
Likelihood ratio bootstrap *p*-value^a^	*p* < .001	*p* < .001	***p* < .001**	*p* < .001
AIC^b^	18774.87	18648.95	**18601.33**	18571.01
BIC^c^	18841.38	18733.60	**18704.11**	18691.92
Lo–Mendell–Rubin-adjusted LRT^d^	*p* < .001	*p* < .001	***p* < .001**	*p* = .46
Entropy^e^	.869	.862	**.781**	.804
Sample proportion class	94, 6	88, 4, 8	**8, 5, 83, 4**	5, 5, 10, 80, 0.3%

a *p <* .01 indicates good fit.

b Lower value indicates better fit.

c Lower value indicates better fit.

d Indicates addition of this class significantly improves fit.

e Value closest to 1 indicates high certainty in classification.

### Associations between social support and affective symptoms

First, positive and negative social support were added to the bifactor model in an SEM framework to test for associations with affective symptoms at each age. As shown in Table 3, when testing for positive and negative social support individually (Model 1) and mutually adjusted (Model 2), positive social support was significantly inversely associated with affective symptoms, and negative social support was significantly associated with affective symptoms, at all three ages (Model 1). We tested for possible interactions of positive and negative social support and sex, which were not statistically significant (*p*-values ranging from .34 to .81); therefore, sex was added as a covariate in Model 3.

**Table 3.** Associations of positive and negative social support at age 53 and affective symptoms at ages 53, 60–64, and 69 years Table 3. long description.

	Model 1^a^			Model 2^b^			Model 3^c^		
	*β*	95% CI	*p*	*β*	95% CI	*p*	*β*	95% CI	*p*
**Age 53**									
Positive social support	**−0.06**	**[−0.09, −0.03]**	**<.001**	**−0.04**	**[−0.07, −0.01]**	**.04**	−0.02	[−0.04, 0.02]	.41
Negative social support	**0.13**	**[0.10, 0.16]**	**<.001**	**0.12**	**[0.09, 0.16]**	**<.001**	**0.12**	**[0.09, 0.16]**	**<.001**
**Age 60–64**									
Positive social support	**−0.13**	**[−0.19, −0.07]**	**<.001**	**−0.08**	**[−0.14, −0.02]**	**.03**	−0.04	[−0.10, 0.02]	.27
Negative social support	**0.19**	**[0.13, 0.24]**	**<.001**	**0.17**	**[0.11, 0.23]**	**<.001**	**0.16**	**[0.10, 0.22]**	**<.001**
**Age 69**									
Positive social support	**−0.13**	**[−0.19, −0.07]**	**.001**	**−0.11**	**[−0.17, −0.04]**	**.01**	−0.07	[−0.14, −0.00]	.08
Negative social support	**0.12**	**[0.05, 0.18]**	**.003**	**0.09**	**[0.02, 0.15]**	**.04**	**0.09**	**[0.02, 0.16]**	**.03**

*Note:* *β* represents the change in affective symptoms in standard deviations following 1 standard deviation increase in positive/negative social support; statistically significant results are bold.

a Model includes each social support scale individually.

b Model includes positive and negative social support mutually adjusted.

c Model additionally adjusted for covariates: sex, nominated close person, marital status, disability/illness, education, social class, and previous mental health problems.

Adjusted for all covariates, positive social support was no longer associated with affective symptoms at any age; however, negative social support remained significantly associated with affective symptoms at ages 53, 60–64, and 69. Among all the covariates included, only sex and previous mental health at age 36 years showed effects at all three ages. In addition, there were significant effects of the nominated close person and disability/illness at age 53 and education at age 69 (Supplementary Appendix C). The pattern of results remained similar when the analysis was run with the imputed datasets (Supplementary Appendix E), except for the association of positive social support with affective symptoms at age 53 (*β* = −0.02. 95% CI: [−0.04, 0.00], *p* = .04).

Covariates were added to the GMM using the R3STEP approach to test associations between positive and negative social support and affective symptom trajectories, mutually adjusted (Model 2), and fully adjusted for all covariates (Model 3). In Model 2, neither positive nor negative social support was associated with the ‘low and increasing’ trajectory. However, negative social support was significantly associated with the ‘consistently moderate/high’ (OR = 1.54, 95% CI: 1.33, 1.79, *p* < .001) and with ‘moderate/high and decreasing’ (OR = 1.23, 95% CI: 1.04, 1.46, *p =* .02) trajectories. Whereas positive social support showed a protective effect for ‘consistently moderate/high’ (OR = 0.79, 95% CI: 0.69, 0.91, *p* = .03) but not for ‘moderate/high and decreasing’ trajectory (OR = 1.10, 95% CI: 0.96, 1.27, *p* = .16). Interactions with sex and positive and negative support were not statistically significant (*p*-values ranging from .16 to .79), therefore sex was added as a covariate in Model 3.

In Model 3, associations remained significant between negative social support and the ‘consistently moderate/high’ (OR = 1.65, 95% CI: 1.36, 2.01, *p* < .001) but not the ‘moderate/high and decreasing’ trajectory (OR = 1.20, 95% CI: 0.96, 1.51, *p* = .11). Positive social support was no longer significantly associated with the ‘consistently moderate/high’ trajectory (OR = 0.84, 95% CI: 0.64, 1.09, *p* = .19) (Supplementary Appendix D). The pattern of results remained similar when the analysis was run with the imputed datasets (Supplementary Appendix F) (Table 4).

**Table 4.** Associations of positive and negative social support and affective symptom trajectories (reference category: no/low symptoms) Table 4. long description.

	Model 1^a^			Model 2^b^			Model 3^c^		
	OR	95% CI	*p*	OR	95% CI	*p*	OR	95% CI	*p*
**Low and increasing**									
Positive social support	0.94	[0.80, 1.02]	.14	0.94	[0.87, 1.03]	.19	0.96	[0.85, 1.08]	.48
Negative social support	1.04	[0.93, 1.13]	.72	1.04	[0.92, 1.16]	.54	1.06	[0.92, 1.23]	.43
**Consistently moderate/high**									
Positive social support	**0.72**	**[0.64, 0.82]**	**<.001**	**0.79**	**[0.69, 0.91]**	**.03**	0.84	[0.64, 1.09]	.19
Negative social support	**1.62**	**[1.40, 1.86]**	**<.001**	**1.54**	**[1.33, 1.79]**	**<.001**	**1.65**	**[1.36, 2.01]**	**<.001**
**Moderate/high and decreasing**									
Positive social support	1.04	[0.93, 1.20]	.52	1.10	[0.96, 1.27]	.16	1.14	[0.93, 1.38]	.20
Negative social support	**1.21**	**[1.02, 1.43]**	**.03**	**1.23**	**[1.04, 1.46]**	**.02**	1.20	[0.96, 1.51]	.11

*Note*: Statistically significant results are bold.

a Model includes each social support scale individually.

b Model includes positive and negative social support mutually adjusted.

c Model additionally adjusted for covariates: sex, marital status, disability/illness, previous mental health problems, education, and social class.

## Discussion

In this study, we investigated the effect of positive and negative social support on affective symptoms at individual time points over a 16-year period and on affective symptom trajectories from midlife (age 53) to later life (age 69). We identified four distinct trajectories of affective symptoms: ‘no/low’, ‘low and increasing’, ‘consistently moderate/high’, and ‘moderate/high and decreasing’. These findings align with a systematic review of general population studies (5–23 years follow-up) showing that three to four trajectories of differing severity and stability are most commonly identified (Musliner, Munk-Olsen, Eaton, & Zandi, 2016). Similar to the current study, the majority of studies in this review reported the ‘low and stable’ group as the largest group and ‘consistently high symptoms’ as a smaller but frequently reported group (Musliner, Munk-Olsen, Eaton, & Zandi, 2016). Thompson, Ploubidis, Richards, and Gaysina (2022) investigated affective symptoms across the life course in the NSHD cohort and similarly found that most individuals had no/low symptoms from midlife into older age; however, they reported a larger proportion (25%) following decreasing symptom trajectories, whereas this group was small in the current study. This may reflect differences in methodological approaches and time periods used to derive latent factor scores and trajectories. More broadly, while midlife is often described as a nadir in mental health at the population level, this is typically based on mean-level trends. In our nationally representative sample, the majority of individuals reported low or stable levels of affective symptoms across time, leaving a relatively small subgroup with scope for marked improvement. Growth mixture modeling captures heterogeneity in trajectories rather than average change, meaning that population-level improvements may reflect modest shifts across a large stable group rather than a distinct ‘improving’ class (Jung & Wickrama, 2008). As such, the small size of the improving group is not inconsistent with prior findings.

Turning to the association of quality of social support with affective symptoms, we demonstrated that negative social support was associated with affective symptoms at all ages and with the ‘consistently moderate/high symptoms’ trajectory. Positive social support was not associated with affective symptoms at individual time points nor with any of the affective symptom trajectories. The lack of findings for positive social support is contrary to previous research, which reported a significant association between positive social support and the likelihood of depression at follow-up (Stafford, McMunn, Zaninotto, & Nazroo, 2011; Thomas, 2016). One explanation for this could be that our study investigated the effect of positive social support over a longer period; it is conceivable that the quality of such support may change during/after midlife. Additionally, the current study tested the social support received from one nominated close person, and it may be that diversity of social support is important in protecting against affective symptoms (Andersen et al., 2021; Li et al., 2021; Turner et al., 2022).

Our finding on negative social support was in line with previous literature, which consistently reported negative social support to be a risk factor for affective symptoms (Hsu, 2012; Kuchibhatla, Fillenbaum, Hybels, & Blazer, 2012; Stafford, McMunn, Zaninotto, & Nazroo, 2011; Teo, Choi, & Valenstein, 2013). In particular, negative social support was significantly associated with the ‘consistently moderate/high symptoms’ but not the ‘low and increasing’ or ‘high and decreasing’ trajectories. This may suggest a complex bidirectional relationship between negative social support and affective symptoms: those experiencing negative social support may experience more affective symptoms but those with higher affective symptoms may perceive social support to be lacking or feel less socially connected, or feel less socially connected as a consequence of being socially rejected due to expressing high levels of negative affect (Schwartz & Litwin, 2019; Turner et al., 2022). In turn, this may lead to persistent affective symptoms, which is in line with our current findings, and may indicate a vicious cycle whereby negative social support may contribute to affective symptoms, and expressing negative affective symptoms may contribute to social rejection and so on. Negative social support was not associated with the ‘low and increasing symptoms’ trajectory. These findings correspond with Kuchibhatla, Fillenbaum, Hybels, and Blazer (2012), who reported no significant association of social support factors and the likelihood of falling into the ‘decliner’ class, and Hsu (2012), who reported social support to be significantly associated with ‘low and increasing symptoms’ for men, but not women. However, Chou (2007) reported that negative family exchanges had a significant effect on the development of depression in those who did not report depression at baseline. In this study, however, the follow-up was much shorter (2 years), and social support was investigated in the presence of pain, which may either indicate more proximal effects of social support, or that (lack of) social support may play a role, especially in combination with other stressors. This would be an interesting avenue for future research. Taken together, these results suggest that negative social support is associated with continued affective symptoms from midlife to later life.

A key strength of this study is the longitudinal exploration of affective symptoms over two decades from midlife into older age, in a large, nationally representative sample. Additionally, multiple repeated and consistent assessments of affective symptoms over time allowed for the modeling of longitudinal trajectories, using psychometrically derived latent factor scores. A further strength is the use of the three-step (R3STEP) approach to estimate covariate-class associations, which accounts for classification uncertainty in latent class membership. However, there are some noteworthy limitations. One of the main challenges with any longitudinal data is attrition and missing data. From the original sample of 5,362 participants, complete information was available for 1,948 participants. Missing data were explored by comparing the sample with and without missing data on key variables. In addition, missing data have been addressed by using full information maximum likelihood and multiple imputation techniques. To determine whether the method used to account for missing data affected the results, the fully adjusted models were rerun with the imputed dataset. The results remained largely the same, but showed a trend for positive social support, which was associated with affective symptoms at age 53 and a lower likelihood of ‘consistently moderate/high’ affective symptoms.

The GMM identifying affective symptom trajectories was restricted to linear trajectories due to only three time points being included in this study. It is possible that affective symptom trajectories may be non-linear, for example, U-shaped or inverse U-shaped patterns; however, a previous systematic review of longitudinal trajectory research for depression reported these to be rare (Musliner, Munk-Olsen, Eaton, & Zandi, 2016).

The measure of social support used in this study was limited to one nominated close person, which may overlook other important relationships presenting buffering or exacerbating effects, and therefore, future research may wish to include measures of the quality of all social relationships (Andersen et al., 2021; Turner et al., 2022). Lastly, it would be interesting to expand the current study to include additional risk and protective factors and investigate potential cumulative, interacting, or buffering effects (Su et al., 2022).

In conclusion, this study identified negative social support as an important predictor for persistent moderate to high affective symptoms. The findings show that individuals differ in their progression of affective symptoms from midlife and can help inform public health or clinical intervention and prevention initiatives by highlighting the importance of modifiable risk factors, such as negative social support (Umberson & Karas Montez, 2010). Addressing close interpersonal relationships in psychological treatments such as Interpersonal Psychotherapy (IPT) has demonstrated efficacy (Cuijpers et al., 2011; Lipsitz & Markowitz, 2013). Interventions may also expand to the wider family network, including carers, to ensure effective psychological support for both the person affected by mental health issues and their carers (Mbedzi, van der Wath, & Moagi, 2025) or to directly address the hostile or critical behavior of other family members (Chambless, 2012) to ultimately help ameliorate persistent affective symptoms and improve quality of life in older age.

## Supporting information

10.1017/S0033291726104930.sm001 Schmidt et al. supplementary material Schmidt et al. supplementary material

## Supplementary material

The supplementary material for this article can be found at http://doi.org/10.1017/S0033291726104930.

## Long descriptions

### Table 1. Long description

The table contains four columns: Variable, Level, Complete cases sample, and Imputed sample.

* Positive social support mean S D: 6.47 1.81 for complete; 6.43 1.87 for imputed.

* Negative social support mean S D: 1.80 1.61 for complete; 1.83 1.64 for imputed.

* Sex: Male is 50.4 percent complete, 49.8 percent imputed; Female is 49.6 percent complete, 50.2 percent imputed.

* Nominated close person: Spouse or partner is the highest category at 84.6 percent complete and 82.9 percent imputed. Other categories include Son or daughter, Other relative, Friend or neighbor, and Other.

* Education: No qualification is the largest group at 46.4 percent complete and 47.8 percent imputed. Other levels include Below ordinary secondary, Ordinary secondary, Advanced secondary, and Higher education.

* Marital status: Married or cohabiting is the majority at 80.5 percent complete and 78.1 percent imputed. Other levels include Single, Married or separated, Divorced, and Widowed.

* Illness: No is 94.1 percent complete and 93.9 percent imputed; Yes is 5.9 percent complete and 6.1 percent imputed.

* Previous mental health problems mean S D: At Age 15, 0.66 1.00 complete and 0.73 1.07 imputed; At Age 36, 2.18 3.52 complete and 2.34 3.69 imputed.

* Affective symptoms trajectories: No or low symptoms is the most common at 83.0 percent complete and 83.2 percent imputed. Other trajectories include Low and increasing, Consistently moderate or high, and Moderate or high and decreasing.

Navigate back to Table 1..

### Table 2. Long description

The table consists of 5 columns and 7 rows. The columns are labeled: Index Type, 2 classes, 3 classes, 4 classes, and 5 classes.

* Likelihood ratio bootstrap p-value: p is less than .001 for all classes (2, 3, 4, and 5).

* A I C: 18774.87 for 2 classes, 18648.95 for 3 classes, 18601.33 for 4 classes, and 18571.01 for 5 classes.

* B I C: 18841.38 for 2 classes, 18733.60 for 3 classes, 18704.11 for 4 classes, and 18691.92 for 5 classes.

* Lo-Mendell-Rubin-adjusted L R T: p is less than .001 for 2, 3, and 4 classes, but p equals .46 for 5 classes.

* Entropy: .869 for 2 classes, .862 for 3 classes, .781 for 4 classes, and .804 for 5 classes.

* Sample proportion class: 94 and 6 for 2 classes; 88, 4, and 8 for 3 classes; 8, 5, 83, and 4 for 4 classes; 5, 5, 10, 80, and 0.3 percent for 5 classes.

The 4 classes column is bolded across all indices, indicating it is the preferred model based on the Lo-Mendell-Rubin-adjusted L R T significance and other fit criteria.

Navigate back to Table 2..

### Figure 1. Long description

The horizontal X axis represents Age with three markers at 53, 60 to 64, and 69. The vertical Y axis represents Affective symptoms latent factor scores ranging from negative 0.75 to 1.5.

Four data series are plotted.

1. No forward slash low symptoms. Represented by a black line with square markers that remains nearly flat and stable just below the 0 line across all ages.

2. Low and increasing. Represented by a dark gray line with diamond markers starting near negative 0.1 at age 53, rising to 0.4 at age 60 to 64, and peaking at approximately 1.3 at age 69.

3. High and decreasing. Represented by a medium gray line with square markers starting at 0.9 at age 53, dropping to 0.1 at age 60 to 64, and ending at negative 0.65 at age 69.

4. Consistently high. Represented by a dark gray line with circular markers starting at 1.3 at age 53 and showing a gradual linear decrease to 0.7 at age 69.

Navigate back to Figure 1..

### Table 3. Long description

The table is organized into three main age-based sections, each evaluating positive and negative social support across three models. Each model reports a coefficient b, a 95 percent C I, and a p-value.

* Age 53 section:

- Positive social support: Model 1 b equals negative 0.06 (p less than .001); Model 2 b equals negative 0.04 (p equals .04); Model 3 b equals negative 0.02 (p equals .41).

- Negative social support: Model 1 b equals 0.13 (p less than .001); Model 2 b equals 0.12 (p less than .001); Model 3 b equals 0.12 (p less than .001).

* Age 60 to 64 section:

- Positive social support: Model 1 b equals negative 0.13 (p less than .001); Model 2 b equals negative 0.08 (p equals .03); Model 3 b equals negative 0.04 (p equals .27).

- Negative social support: Model 1 b equals 0.19 (p less than .001); Model 2 b equals 0.17 (p less than .001); Model 3 b equals 0.16 (p less than .001).

* Age 69 section:

- Positive social support: Model 1 b equals negative 0.13 (p equals .001); Model 2 b equals negative 0.11 (p equals .01); Model 3 b equals negative 0.07 (p equals .08).

- Negative social support: Model 1 b equals 0.12 (p equals .003); Model 2 b equals 0.09 (p equals .04); Model 3 b equals 0.09 (p equals .03).

Model 1 includes scales individually. Model 2 is mutually adjusted for both support types. Model 3 adds adjustments for sex, marital status, disability, education, social class, and previous mental health.

Navigate back to Table 3..

### Table 4. Long description

The table presents O R, 95 percent C I, and p-values for three models.

1. Low and increasing trajectory:

- Positive social support: O R ranges from 0.94 to 0.96 across models, with p-values from .14 to .48.

- Negative social support: O R ranges from 1.04 to 1.06 across models, with p-values from .43 to .72.

2. Consistently moderate/high trajectory:

- Positive social support: Significant O R of 0.72 (p less than .001) in Model 1 and 0.79 (p equals .03) in Model 2. Model 3 O R is 0.84 (p equals .19).

- Negative social support: Highly significant across all models. O R is 1.62 in Model 1, 1.54 in Model 2, and 1.65 in Model 3 (all p less than .001).

3. Moderate/high and decreasing trajectory:

- Positive social support: O R ranges from 1.04 to 1.14 across models, with p-values from .16 to .52.

- Negative social support: Significant O R of 1.21 (p equals .03) in Model 1 and 1.23 (p equals .02) in Model 2. Model 3 O R is 1.20 (p equals .11).

Footnotes indicate Model 1 includes scales individually, Model 2 is mutually adjusted, and Model 3 adjusts for sex, marital status, disability, previous mental health, education, and social class.

Navigate back to Table 4..
