# Supplementary material for: Associations of quality of social support with affective symptoms from midlife to later life: evidence from a British birth cohort
Source: Psychol Med. 2026 Jul 9;56:e223. doi: 10.1017/S0033291726104930 (PMC13370177; doi:10.1017/S0033291726104930)
Supplement: Schmidt et al. supplementary material [file S0033291726104930sup001.docx]

**Appendices**

### Appendix A

*CFA factor structure fit indices (Bifactor model)*

|  | **Age 53*** | **Age 60-64*** | **Age 69*** |
| --- | --- | --- | --- |
| **c^2^** | 3042.05, p < .001 | 2051.39, p <.001 | 2324.64, p < .001 |
| **RMSEA** | 0.054 | 0.049 | 0.054 |
| **CFI** | 0.970 | 0.966 | 0.960 |
| **TLI** | 0.964 | 0.959 | 0.953 |
| **SRMR** | 0.051 | 0.054 | 0.061 |

*These models include additional covariances between highly correlated items 5 and 6 and items 8 and 9.

###

### Appendix B

*Measurement invariance indices for GHQ over time*

|  | **χ2 Model fit** | **χ2 difference test** | **RMSEA** | **CFI** | **TLI** | **SRMR** |
| --- | --- | --- | --- | --- | --- | --- |
| Configural (1) | χ2 (3159) = 10424.05,  p < .001 | -- | .027 | .952 | .947 | .054 |
| Metric | Not applicable | | | | | |
| Scalar  (2) | χ2 (3323) = 10209.30,  p < .001 | χ2 (164) = 598.55,  p < .001 | .026 | .955 | .952 | .056 |

*Reference model

Model 1: Unconstrained model (reference);

Model 2: Factor loadings and thresholds constrained to be equal across time points

### Appendix C

*Associations of positive and negative social support with affective symptoms at ages 53, 60-64 and 69, fully adjusted*

|  | **Model 3** | | |
| --- | --- | --- | --- |
|  | **b** | **95% CI** | ***p*** |
| **Age 53** |  |  |  |
| Positive social support | -0.02 | [-0.04, 0.02] | .41 |
| Negative social support | **0.12** | **[0.09, 0.16]** | **<.001** |
| Sex | **0.30** | **[0.21, 0.37]** | **<.001** |
| Marital status | 0.03 | [-0.03, 0.08] | .38 |
| Nominated close person | **0.03** | **[0.01, 0.05]** | **.04** |
| Illness/disability | **0.84** | **[0.68, 1.01]** | **<.001** |
| Education | 0.00 | [-0.03, 0.04] | .84 |
| Social class | 0.01 | [-0.03, 0.05] | .69 |
| Previous mental health – age 15 | 0.00 | [-0.03. 0.04] | .85 |
| Previous mental health – age 36 | **0.06** | **[0.05, 0.07]** | **<.001** |
| **Age 60-64** |  | | |
| Positive social support | -0.04 | [-0.10, 0.02] | .27 |
| Negative social support | **0.16** | **[0.10, 0.22]** | **<.001** |
| Sex | **0.10** | **[0.06, 0.15]** | **<.001** |
| Marital status | 0.00 | [-0.05, 0.06] | .90 |
| Nominated close person | -0.02 | [-0.07, 0.03] | .51 |
| Illness/disability | 0.05 | [-0.00, 0.09] | .12 |
| Education | -0.03 | [-0.08, 0.03] | .45 |
| Social class | 0.07 | [0.01, 0.12] | .05 |
| Previous mental health – age 15 | 0.03 | [-0.01, 0.08] | .25 |
| Previous mental health – age 36 | **0.24** | **[0.19, 0.28]** | **<.001** |
| **Age 69** |  | | |
| Positive social support | -0.07 | [-0.15, -0.01] | .08 |
| Negative social support | **0.09** | **[0.03, 0.16]** | **.03** |
| Sex | **0.10** | **[0.05, 0.15]** | **.002** |
| Marital status | -0.01 | [-0.06, 0.05] | .81 |
| Nominated close person | 0.03 | [-0.02, 0.09] | .29 |
| Illness/disability | 0.06 | [0.01, 0.10] | .05 |
| Education | **-0.10** | **[-0.15, -0.04]** | **.01** |
| Social class | 0.02 | [-0.04, 0.08] | .52 |
| Previous mental health – age 15 | 0.03 | [-0.02, 0.08] | .32 |
| Previous mental health – age 36 | **0.19** | **[0.15, 0.24]** | **<.001** |

### Appendix D

*Associations of positive and negative social support with affective symptom trajectory classes (no/low affective symptoms is the reference group), fully adjusted (N = 1,948), using the R3 step approach*

|  |  | **OR** | **(95% CI)** | **p** |
| --- | --- | --- | --- | --- |
| **AS trajectories** |  |  |  |  |
| **Low and increasing** |  |  |  |  |
| Positive social support |  | 0.96 | (0.85, 1.08) | .48 |
| Negative social support |  | 1.06 | (0.92, 1.23) | .43 |
| Sex |  | **1.57** | **(1.00, 2.47)** | **<.05** |
| Marital status |  | 0.99 | (0.73, 1.34) | .93 |
| Nominated close person |  | 1.01 | (0.89, 1.15) | .86 |
| Illness/Disability |  | 0.76 | (0.20, 2.88) | .69 |
| Education |  | 0.99 | (0.84, 1.17) | .91 |
| Social class |  | 0.98 | (0.80, 1.20) | .85 |
| Previous mental health problems | Age 15 | 1.06 | (0.85, 1.32) | .63 |
|  | Age 36 | **1.09** | **(1.03, 1.15)** | **.01** |
| **Consistently moderate/high** |  |  |  |  |
| Positive social support |  | 0.84 | (0.64, 1.09) | .19 |
| Negative social support |  | **1.65** | **(1.36, 2.01)** | **<.001** |
| Sex |  | **2.23** | (0.83, 6.00) | .11 |
| Marital status |  | 1.24 | (0.77, 1.98) | .38 |
| Nominated close person |  | 1.08 | (0.90, 1.29) | .43 |
| Illness/Disability |  | **8.02** | **(2.22, 28.97)** | **.00** |
| Education |  | 0.93 | (0.63, 1.38) | .72 |
| Social class |  | **1.49** | **(1.04, 2.13)** | **.03** |
| Previous mental health problems | Age 15 | 0.98 | (0.63, 1.51) | .91 |
|  | Age 36 | **1.22** | **(1.13, 1.31)** | **<.001** |
| **Moderate/high and decreasing** |  |  |  |  |
| Positive social support |  | 1.14 | (0.93, 1.38) | .20 |
| Negative social support |  | 1.20 | (0.96, 1.51) | .11 |
| Sex |  | 2.19 | (1.19, 4.05) | .08 |
| Marital status |  | 1.21 | (0.82, 1.78) | .34 |
| Nominated close person |  | 0.76 | (0.56, 1.03) | .46 |
| Illness/Disability |  | **2.73** | **(1.04, 7.22)** | **.04** |
| Education |  | **1.40** | **(1.07, 1.82)** | **.02** |
| Social class |  | 1.08 | (0.79, 1.47) | .65 |
| Previous mental health problems | Age 15 | 0.96 | (0.75, 1.22) | .86 |
|  | Age 36 | **1.11** | **(1.03, 1.20)** | **.01** |

### Appendix E

*Associations of positive and negative social support with affective symptoms at ages 53, 60-64 and 69, fully adjusted with imputed sample*

|  | **Model 3** | | |
| --- | --- | --- | --- |
|  | **b** | **95% CI** | ***p*** |
| **Age 53 (n = 2901)** |  |  |  |
| Positive social support | **-0.02** | **[-0.04, 0.00]** | **.04** |
| Negative social support | **0.12** | **[0.10, 0.15]** | **.032** |
| Sex | **0.30** | **[0.23, 0.36]** | **.012** |
| Marital status | **0.00** | **[-0.04, 0.04]** | **.023** |
| Nominated close person | 0.07 | [0.03, 0.11] | .09 |
| Illness/disability | **0.73** | **[0.60, 0.86]** | **.005** |
| Education | -0.02 | [-0.05, 0.01] | .07 |
| Social class | 0.00 | [-0.03, 0.03] | .13 |
| Previous mental health – age 15 | 0.02 | [-0.02. 0.05] | .14 |
| Previous mental health – age 36 | 0.06 | [0.05, 0.07] | .08 |
| **Age 60-64 (n = 2190)** |  | | |
| Positive social support | -0.02 | [-0.10, 0.02] | .11 |
| Negative social support | 0.10 | [0.10, 0.22] | .08 |
| Sex | **0.22** | **[0.06, 0.15]** | **.02** |
| Marital status | -0.01 | [-0.05, 0.06] | .09 |
| Nominated close person | -0.02 | [-0.07, 0.03] | .51 |
| Illness/disability | **0.15** | **[-0.00, 0.09]** | **.04** |
| Education | -0.03 | [-0.08, 0.03] | .07 |
| Social class | 0.06 | [0.01, 0.12] | .12 |
| Previous mental health – age 15 | 0.04 | [-0.01, 0.08] | .16 |
| Previous mental health – age 36 | 0.08 | [0.19, 0.28] | .08 |
| **Age 69 (n = 2125)** |  | | |
| Positive social support | -0.03 | [-0.06, -0.00] | .13 |
| Negative social support | 0.09 | [0.06, 0.12] | .07 |
| Sex | **0.20** | **[0.12, 0.28]** | **.02** |
| Marital status | **-0.03** | **[-0.08, 0.02]** | **<.05** |
| Nominated close person | 0.03 | [-0.03, 0.09] | .12 |
| Illness/disability | 0.21 | [0.03, 0.38] | .08 |
| Education | -0.07 | [-0.10, -0.03] | .07 |
| Social class | 0.05 | [0.02, 0.09] | .13 |
| Previous mental health – age 15 | 0.06 | [0.02, 0.10] | .15 |
| Previous mental health – age 36 | 0.06 | [0.05, 0.07] | .17 |

### Appendix F

*Associations of positive and negative social support with affective symptom trajectory classes (no/low affective symptoms is the reference group), fully adjusted, with imputed sample*

*(N = 3,121), using the R3 step approach*

|  |  | **OR** | **(95% CI)** | ***p*** |
| --- | --- | --- | --- | --- |
| **AS trajectories** |  |  |  |  |
| **Low and increasing** |  |  |  |  |
| Positive social support |  | 0.95 | (0.86, 1.04) | .25 |
| Negative social support |  | 1.02 | (0.91, 1.15) | .72 |
| Sex |  | 1.38 | (0.98, 1.94) | .07 |
| Nominated close person |  | 0.98 | (0.88, 1.09) | .69 |
| Illness/Disability |  | 1.11 | (0.49, 2.52) | .80 |
| Education |  | 0.98 | (0.85, 1.12) | .74 |
| Social class |  | 1.00 | (0.85, 1.18) | .97 |
| Previous mental health problems | Age 15 | 1.08 | (0.91, 1.29) | .38 |
|  | Age 36 | **1.10** | **(1.05, 1.15)** | **<.001** |
| **Consistently moderate/high** |  |  |  |  |
| Positive social support |  | **0.81** | **(0.68, 0.97)** | **.02** |
| Negative social support |  | **1.54** | **(1.31, 1.81)** | **<.001** |
| Sex |  | **2.39** | **(1.24, 4.61)** | **.01** |
| Nominated close person |  | 1.07 | (0.93, 1.24) | .35 |
| Illness/Disability |  | **7.57** | **(2.98, 19.19)** | **<.001** |
| Education |  | 0.89 | (0.66, 1.20) | .44 |
| Social class |  | 1.28 | (0.98, 1.69) | .07 |
| Previous mental health problems | Age 15 | 1.13 | (0.81, 1.58) | .47 |
|  | **Age 36** | **1.24** | **(1.16, 1.31)** | **<.001** |

| **Moderate/high and decreasing** |  |  |  |  |
| --- | --- | --- | --- | --- |
| Positive social support |  | 1.07 | (0.92, 1.25) | .36 |
| Negative social support |  | **1.22** | **(1.01, 1.46)** | **.04** |
| Sex |  | **2.28** | **(1.33, 3.91)** | **.00** |
| Nominated close person |  | 0.88 | (0.71, 1.09) | .25 |
| Illness/Disability |  | 1.99 | (0.74, 5.32) | .17 |
| Education |  | 1.25 | (1.00, 1.57) | .05 |
| Social class |  | 1.05 | (0.80, 1.37) | .72 |
| Previous mental health problems | Age 15 | 0.90 | (0.64, 1.27) | .56 |
|  | **Age 36** | **1.11** | **(1.03, 1.19)** | **.01** |

**Appendix G**

*Diagrams of the psychometric model of the GHQ-28 with factor loadings for all three time points:a) age 53, b) age 60-64 and c) age 69.*

1. *Age 53*

**
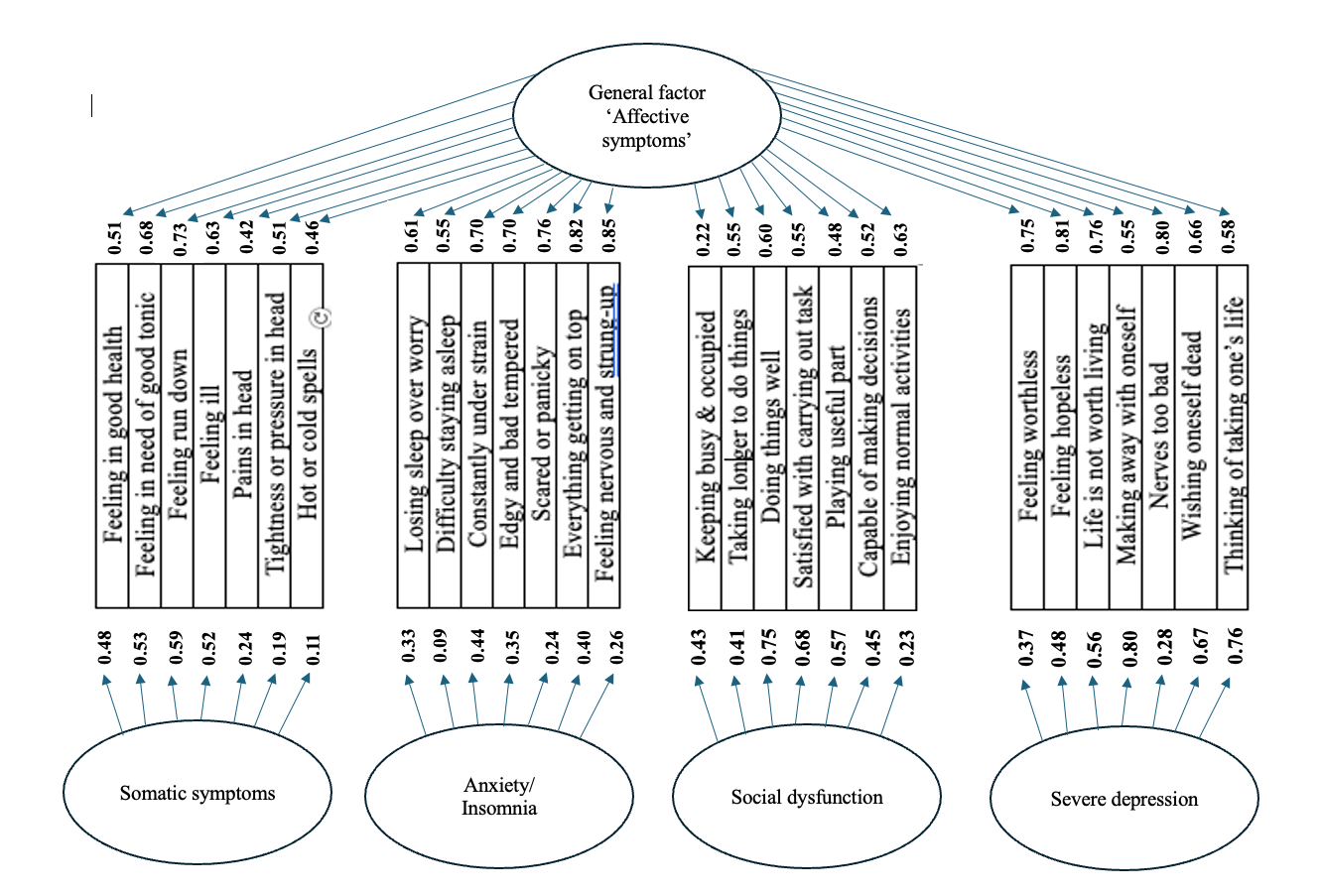
**

1. *Age 60-64*

*
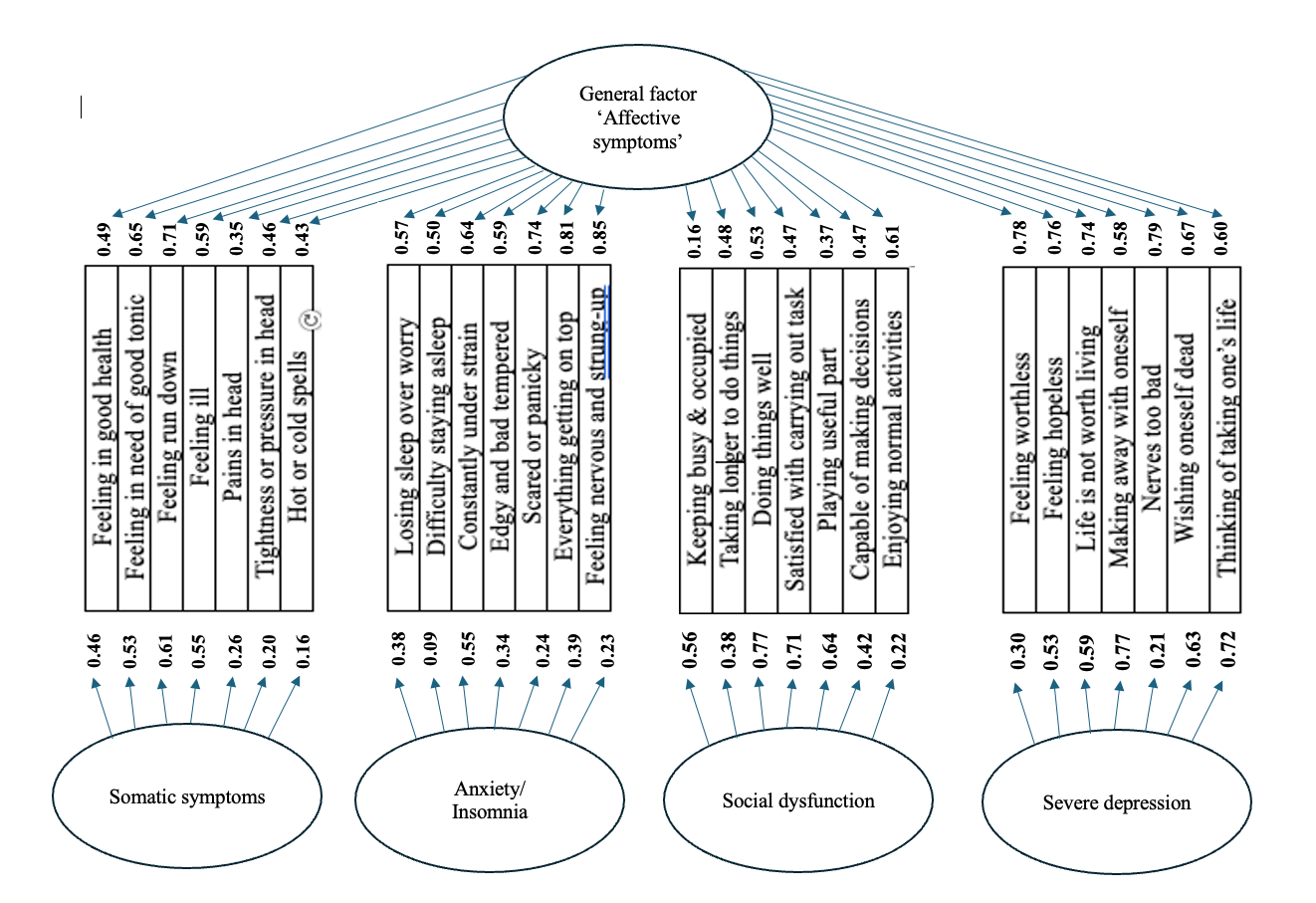
*

*c. Age 69*

*
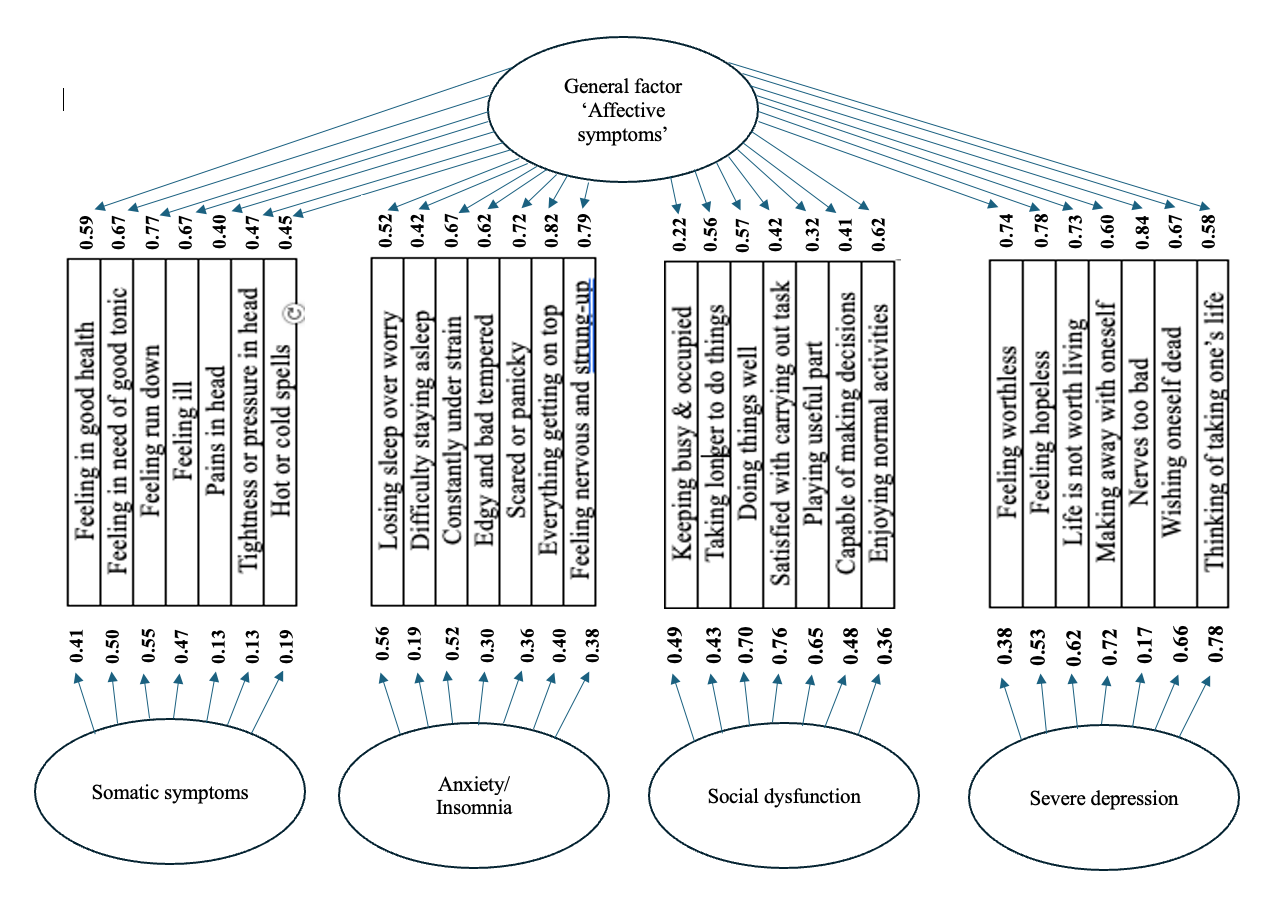
*
